# Pyramidal cell development: postnatal spinogenesis, dendritic growth, axon growth, and electrophysiology

**DOI:** 10.3389/fnana.2014.00078

**Published:** 2014-08-12

**Authors:** Guy N. Elston, Ichiro Fujita

**Affiliations:** ^1^Centre for Cognitive NeuroscienceSunshine Coast, QLD, Australia; ^2^Graduate School of Frontier Biosciences and Center for Information and Neural Networks, Osaka University and National Institute of Communication TechnologySuita, Japan

**Keywords:** synaptogenesis, axon, dendrite, spine, bouton, cortex, human, macaque

## Abstract

Here we review recent findings related to postnatal spinogenesis, dendritic and axon growth, pruning and electrophysiology of neocortical pyramidal cells in the developing primate brain. Pyramidal cells in sensory, association and executive cortex grow dendrites, spines and axons at different rates, and vary in the degree of pruning. Of particular note is the fact that pyramidal cells in primary visual area (V1) prune more spines than they grow during postnatal development, whereas those in inferotemporal (TEO and TE) and granular prefrontal cortex (gPFC; Brodmann's area 12) grow more than they prune. Moreover, pyramidal cells in TEO, TE and the gPFC continue to grow larger dendritic territories from birth into adulthood, replete with spines, whereas those in V1 become smaller during this time. The developmental profile of intrinsic axons also varies between cortical areas: those in V1, for example, undergo an early proliferation followed by pruning and local consolidation into adulthood, whereas those in area TE tend to establish their territory and consolidate it into adulthood with little pruning. We correlate the anatomical findings with the electrophysiological properties of cells in the different cortical areas, including membrane time constant, depolarizing sag, duration of individual action potentials, and spike-frequency adaptation. All of the electrophysiological variables ramped up before 7 months of age in V1, but continued to ramp up over a protracted period of time in area TE. These data suggest that the anatomical and electrophysiological profiles of pyramidal cells vary among cortical areas at birth, and continue to diverge into adulthood. Moreover, the data reveal that the “use it or lose it” notion of synaptic reinforcement may speak to only part of the story, “use it but you still might lose it” may be just as prevalent in the cerebral cortex.

## Introduction

**Figure 1:**
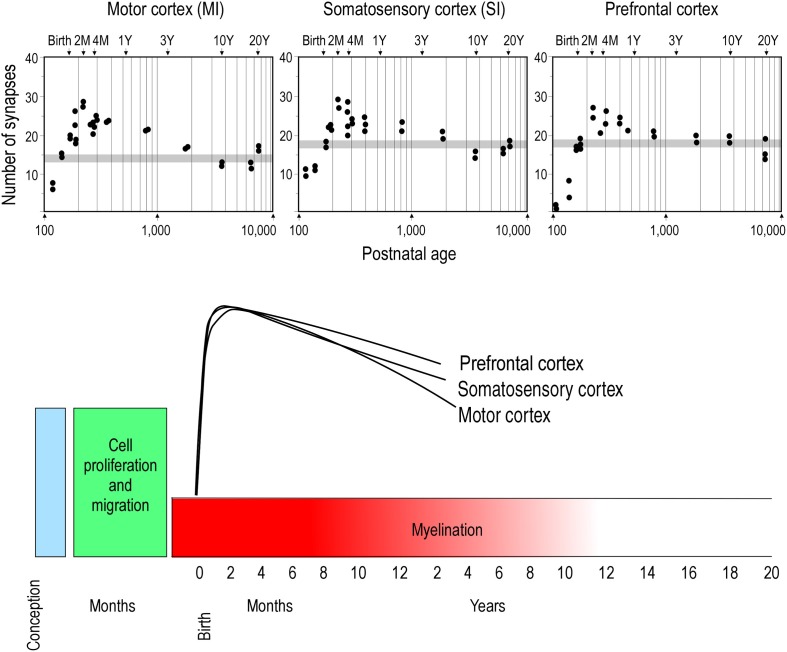
**Concurrent synaptogenesis**. Plots illustrating the density of synapses per 100 μm of cortical neuropil in the primary motor (MI) and somatosensory (SI) areas and ventrolateral prefrontal cortex (Brodmann's area 12) of the macaque monkey. The average synaptic density observed in the adult in each cortical area is represented by gray stipple. Note that in all cortical areas the maximum synapse density is recorded at approximately 2 months of age. Modified from Rakic et al. (1986).

**Figure 2:**
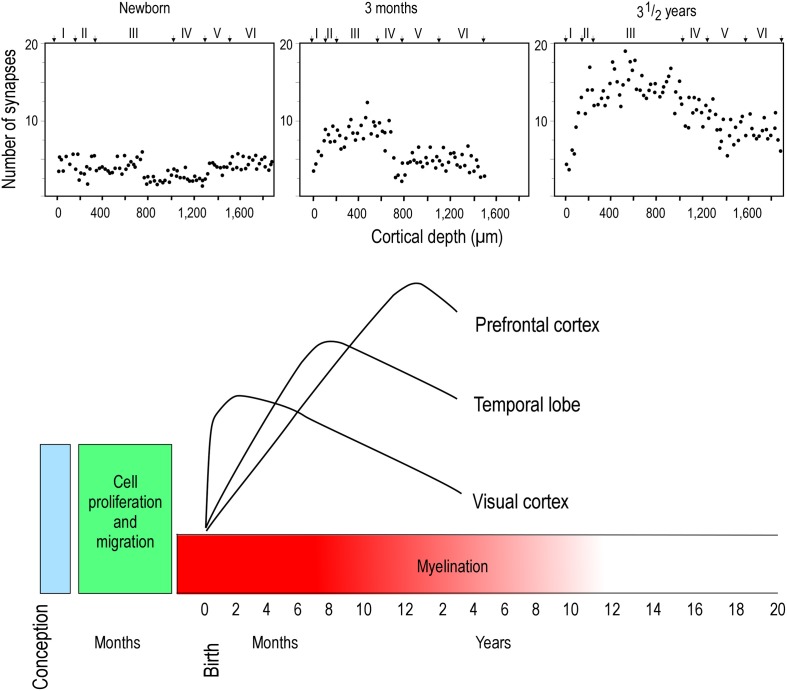
**Hierarchical synaptogenesis**. Plots illustrating the density of synapses per 100 μm of cortical neuropil in human left middle frontal gyrus in the newborn, 3 month old and in the three and a half year old postnatal brain. Note that the maximum synapse density at three and a half years of age is greater than that at 3 months of age. Note also the relative differences in synapse densities in the different cortical layers at the different ages illustrated. Modified from Huttenlocher and Dabholkar (1997).

Two opposing theories on postnatal synaptogenesis have dominated over the past two and a half decades. One theory posits that circuitry in all cortical areas matures concurrently (Rakic et al., 1986), whereas the other states that circuitry in different cortical areas mature at different rates (Huttenlocher and Dabholkar, 1997). Both theories have been derived from electronmicroscopic study of the cortical neuropil. In the former hypothesis, the maximum synaptic connectivity is achieved at approximately three and a half months of age, after which there is a net reduction in the number of synaptic connections in cortex (Figure 1). Data were sampled from visual (V1), somatosensory (SI), motor (M1) and prefrontal cortices. In the latter hypothesis, synaptic density in the neuropil may continue to increase over an extended period of time (Figure 2). Data were sampled from V1, auditory (A1) and granular prefrontal cortex (middle frontal gyrus). On the face of it, these hypotheses appear to be irreconcilable. However, these two theories were based on data obtained from different species, data leading to the former view were obtained from the macaque monkey whereas those supporting the later view were obtained from the human brain. Furthermore, these data were sampled from a select few cortical areas, and were not corrected for cell death, changes in myelination nor cortical growth (see Guillery, 2005 for a review). More recent studies, which accounted for changes in myelination and blood vessels, revealed laminar differences in the developmental profiles of synaptogenesis (Bourgeois et al., 1989; Huttenlocher, 1990; Zecevic and Rakic, 1991; Bourgeois and Rakic, 1993; Granger et al., 1995), but do not reconcile the different theories.

A number of other groups have investigated the issue by using a different approach—that of studying the growth of the dendritic trees of neurons. The results are less clear cut than the above interpretations, and vary with neuron type, cortical layer and cortical area. For example, Lund and colleagues, by studying thalamocortial recipient neurons in V1 of the macaque monkey, concluded that dendritic growth is not concurrent (temporally coincident) in different populations of cells, but, rather, there is a temporal disjoin between neurons in the magno- and parvocellular pathways (Lund and Holbach, 1991). Moreover, the study revealed that the basal dendritic trees of pyramidal cells in V1 may continue to grow up to 9 months of age, despite a general trend for a decrease in the number of dendritic spines, the site of excitatory synaptic inputs, after 2 months of age (Boothe et al., 1979; Mates and Lund, 1983a,b). O'Kusky and Colonnier (1982) reported an increase in neuronal connectivity (as revealed by relative neuronal density, synaptic density and cortical thickness) from birth to 6 months of age in V1 of the macaque monkey, being most pronounced in supragranular layers. Becker et al. (1984) reported progressive dendritic growth and branching in the dendritic trees of supragranular pyramidal cells from conception until 2 years of age in human V1. Data reported at 2 years of age was greater than that reported in adults, but, it is difficult to determine at what age the dendritic trees stopped growing and began to retract to the size reported in the adult. Cupp and Uemura (1980) reported continued dendritic growth in pyramidal cells in the superior frontal gyrus of the macaque monkey up to 28 years of age. Koenderink et al. (1994) suggest continued growth of the dendritic trees of infragranular pyramidal cells up to at least 15 years of age in the human gPFC. Studies in the human and chimpanzee cortex reveal postnatal growth of the dendritic trees of pyramidal cells in the gPFC outstrip those in V1: in both species the dendritic trees of cells in the gPFC are small and sparsely branched at birth compared to those in V1, whereas in the adult they are markedly larger and more branched (Travis et al., 2005; Bianchi et al., 2012, 2013). Despite the variability in the results, the current view is that dendritic trees of some neurons may continue to grow well beyond the peak in synaptogenesis. The variability may reflect the different cortical areas, and species, that have been studied.

**Figure 3:**
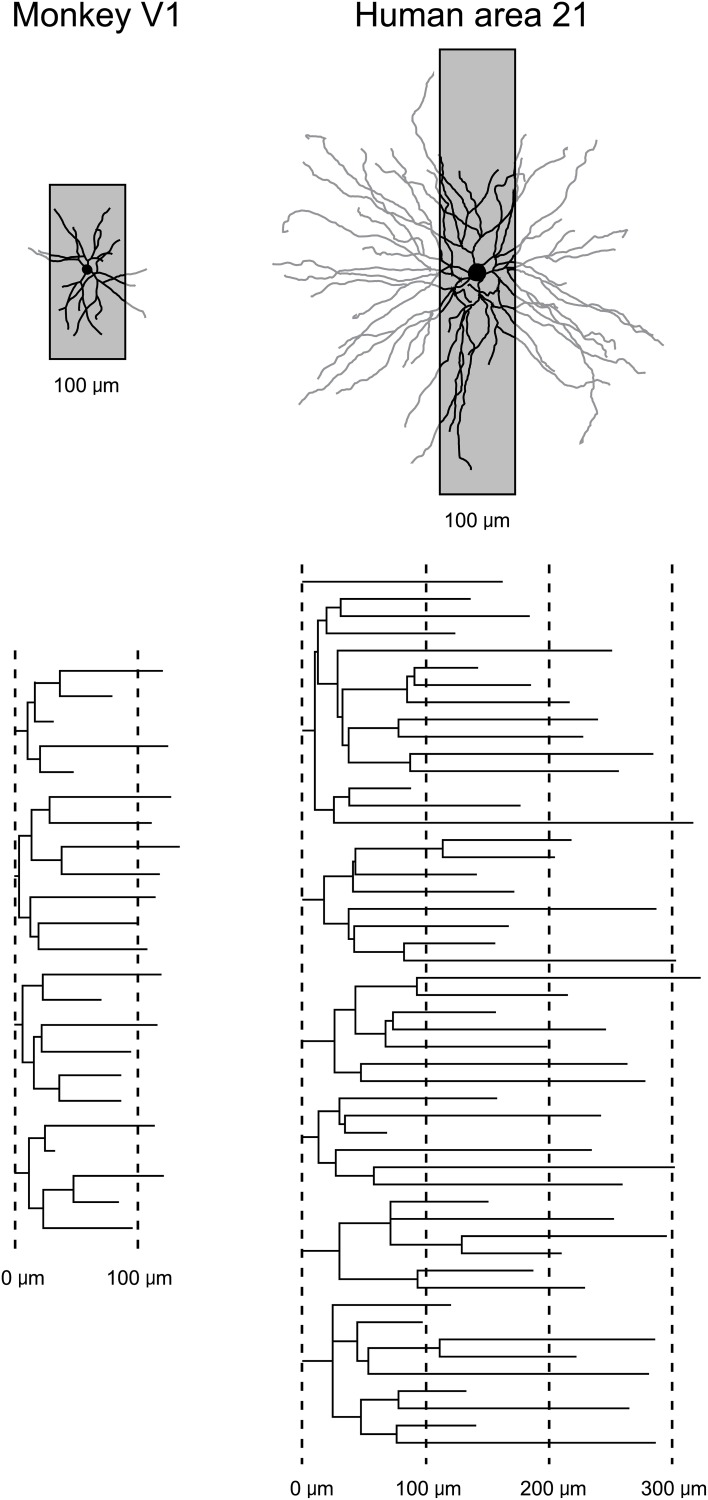
**Transverse vs. tangential plane**. Schematic illustrating how the study of pyramidal cell morphology in the transverse plane may bias for uniformity in structure. Illustrated are two cells sampled from the primary visual area (V1) of the macaque monkey and Brodmann's area 21 of the temporal lobe of human (Brodmann, 1909). The basal dendritic trees of the two cells are shown in the tangential plane. Superimposed on each cell is a 100 μm gray box representing the thickness of section routinely used in Golgi studies. In black is the part of the dendritic tree that would be seen in a Golgi study. In gray is the part of the dendritic tree that would have been clipped off during sectioning and thus invisible to the observer. Below are illustrated the dendrograms of each of the two cells, which resulted from reconstruction of the complete basal dendritic tree as seen in the tangential plane. Based on our observations in the human temporal lobe (Elston et al., 2001), transverse sections used in Golgi studies would have to be > 600 μm thick, and the cell body located in the center of the section, to be able to visualize all dendrites.

While the studies of dendritic growth provide significant insights into how the developmental profiles of neurons may vary among cortical areas, they are difficult to interpret because one must compare data sampled from one cortical area of a given species with another cortical area of a different species, or from one cortical layer with another. In addition, all of these studies relied on the use of Golgi method. As discussed elsewhere (Elston et al., 2011b), this methodology only allows reconstruction of part of the dendritic tree (Figure 3). To visualize the entire basal dendritic tree of pyramidal cells in the human temporal lobe, for example, the sections would have to be at least 600 μm thick. Even then, only neurons with their cell body located some 300 μm into the section could be included for analyses otherwise some dendrites would be clipped (cf. Elston et al., 2001). Thus, while the Golgi method has been used to great effect for more than 100 years and it is fair to say that the field of neuroanatomy was built on this methodology (e.g., refer to the works of Santiago Ramon y Cajal, Karl Retzius, J LeRoy Conel, and Jennifer Lund to mention a few) and is still being used to great effect (e.g., Jacobs et al., 1997, 2001, 2014; Travis et al., 2005; Petanjek et al., 2008; Bianchi et al., 2012, 2013), a different approach was needed to test for area-specific differences in pyramidal cell structure.

## A different way of looking at it

**Figure 4:**
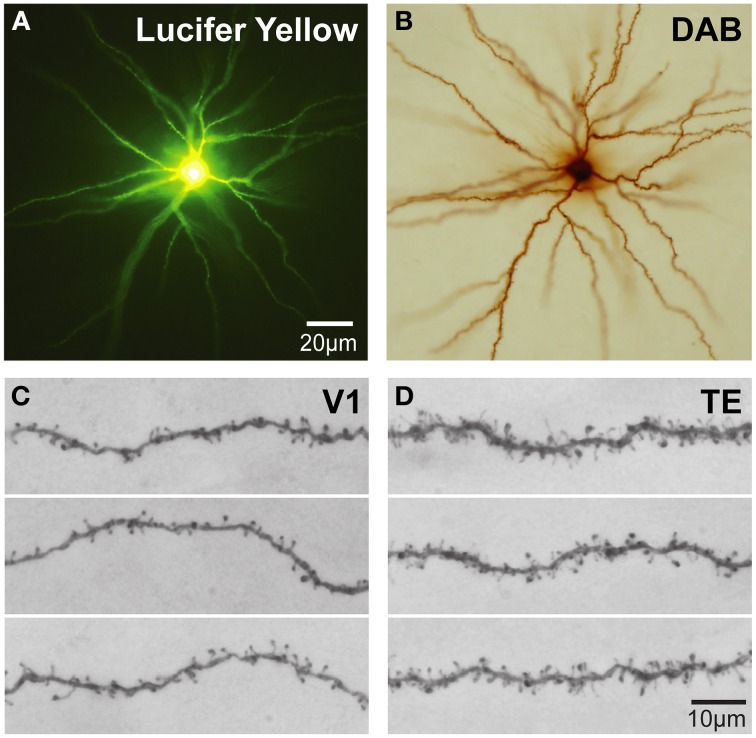
**Cell injection**. **(A)** Low power photomicrographs of Lucifer Yellow-injected layer III pyramidal cells that were injected in tangential sections taken from the granular prefrontal cortex of the macaque monkey (area 12vl) and processed for a DAB reaction product. **(B) (C,D)** Higher power photos of primary visual (V1) and inferotemporal (TE) cortical cells as viewed through a ×100 oil-immersion Zeiss objective, revealing aspects of their fine structure including dendritic spines. Scale bar = 20 μm in **(A,B)** and 10 μm in **(C,D)**.

Intracellular injection of neurons in slices cut tangential to the cortical surface allows the visualization of the entire basal dendritic tree of large numbers of individual cells (>1000) in a single brain allowing an uninterrupted reconstruction of even the longest dendrites (Figure 4) that may project more than 300 μm from the cell body (e.g., Elston et al., 2001) yielding sufficient data to allow robust statistical comparisons. Neurons can be injected randomly, or specific populations of cells can be injected, such as corticocortical projecting or subcortical projecting cells (Vercelli and Innocenti, 1993; Matsubara et al., 1996; Elston and Rosa, 2006). In addition, all of the spines along a dendrite can be visualized with the aid of a high power objective and there is no need to attempt to correct for spines hidden by the Golgi precipitate (e.g., Feldman and Peters, 1979) as the DAB precipitate is less electron dense than the Golgi precipitate and the viewer is able to focus through the DAB precipitate under intense illumination by standard light microscopy (see Elston and Rosa, 1997 for a discussion). Over the past 10 years we have applied this methodology to the study of pyramidal cell development in the cerebral cortex.

## Dendritic growth in postnatal development

**Figure 5:**
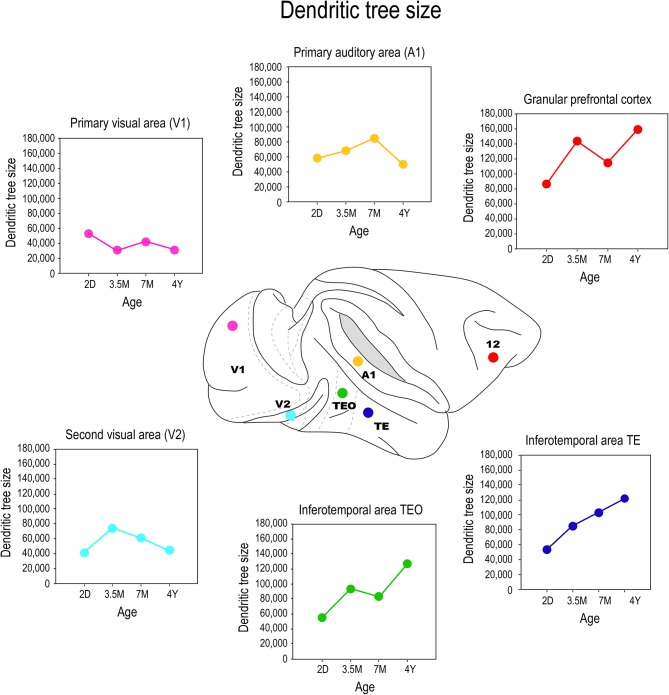
**Growth profiles**. Plots of the size of the basal dendritic trees of layer III pyramidal neurons sampled from primary visual area (V1), the second visual area (V2), the primary auditory cortex (A1), the posterior and anterior portion of the inferotemporal cortex (TEO and TEpd, respectively) and prefrontal cortex (Brodmann's area 12) at postnatal day two (2D), three and a half months of age (3.5M), seven months of age (7M) and four and a half years (4Y) of age. Data taken from Elston et al. (2009, 2010a,b).

The application of the cell injection method in flat-mount sections taken from the macaque monkey revealed systematic differences in the growth profiles of pyramidal cells among cortical areas (Figure 5). Notably, the basal dendritic trees of supragranular pyramidal cells continue to grow into adulthood in high level cortical areas such as visual association areas TEO, and TEpd whereas those in V1 attain their greatest postnatal size around birth (Boothe et al., 1979; Elston et al., 2010a). In granular prefrontal cortex (Brodmann's area 12), the basal dendritic trees of layer III pyramidal cells attain their largest size in adulthood (Elston et al., 2009; see also Cupp and Uemura, 1980; Anderson et al., 1995). The dendritic trees of pyramidal cells in V2 and V4 attain their greatest size at 3.5 months of age, and then decrease in size to their adult type (Elston et al., 2010a). A different profile again has been reported for cells in the primary auditory cortex (A1) in macaque, where cells grow larger dendritic trees from birth until at least 7 months of age, before decreasing in size to their adult form (Elston et al., 2010b).

Comparison of these data with previous Golgi data, and emerging cell injection data in other species, reveals potential species differences in the postnatal growth profiles of pyramidal cells in the cerebral cortex. The trend observed here in pyramidal cell growth in V1 of the macaque monkey is consistent with that reported in human: cells become smaller after birth (Becker et al., 1984). The extended period of growth of the dendritic trees of cells in association areas of the temporal lobe and the granular prefrontal cortex in macaque is consistent with that reported in human (Conel, 1941, 1947, 1955, 1959, 1963, 1967; Elston et al., 2009, 2010a,b, 2011a). However studies in the New World marmoset monkey reveal that pyramidal cells in V1 are larger in the adult than in the newborn (Oga et al., 2013). Clearly, further comparative investigations are required.

## Branching

**Figure 6:**
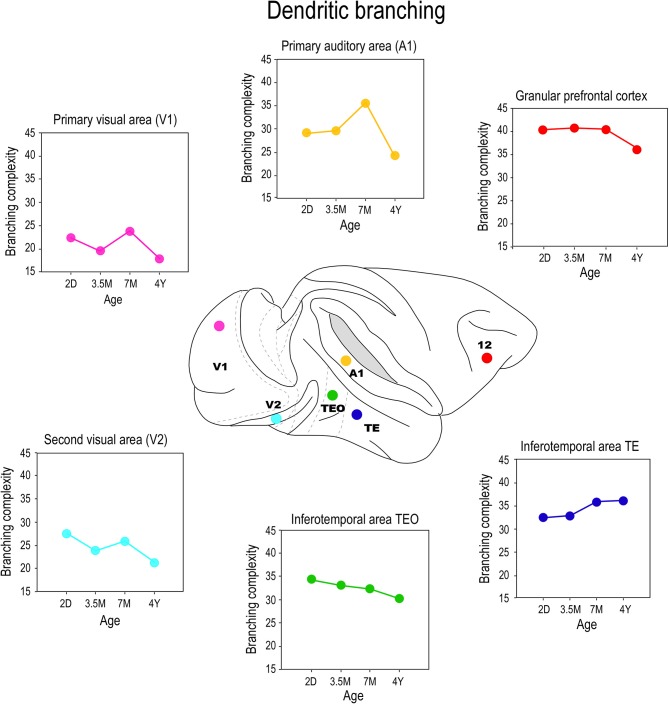
**Branching profiles**. Plots of the branching structure of the basal dendritic trees of layer III pyramidal neurons sampled from primary visual area (V1), the second visual area (V2), the primary auditory cortex (A1), the posterior and anterior portion of the inferior temporal cortex (TEO and TEav, respectively) and prefrontal cortex (Brodmann's area 12) at postnatal day two (2D), three and a half months of age (3.5M), seven months of age (7M) and four and a half years (4Y) of age. Note that both the size and branching structure of the dendritic trees may share similar developmental profiles, or diverge independently (compare Figure 5). Data taken from Elston et al. (2009, 2010a,b, 2011a).

Differences were also observed in the branching profiles of the dendritic trees of pyramidal cells among cortical areas during postnatal development (Figure 6). Contrary to what might be expected from the size, the dendritic trees of layer III pyramidal cells in area TEO of the macaque monkey were most branched at birth, and became less branched through juvenile and adolescent stages into adulthood (Elston et al., 2010a). In V1, V2, and V4, the most branched dendritic trees were observed at PND2 and the least branched dendritic trees were observed in the adult (Elston et al., 2010a). In A1, the dendritic trees of layer III pyramidal cells became increasingly more branched from PND2 to 3.5–7 months of age before declining to their adult values (Elston et al., 2010b). Cells in granular prefrontal area 12, on the other hand, have relatively stable branching structure from birth to adolescence, then become less branched into adulthood, whereas those in area 46 become increasingly more branched from birth to >4.5 years of age (Anderson et al., 1995). While seemingly contradictory, pyramidal cells in different areas within the mature gPFC of the macaque differ in size, branching structure and spine densities (Elston et al., 2011a). In the marmoset monkey, pyramidal cells in V1, TE, and area 12 are more branched in the adult than in the newborn (Oga et al., 2013).

These data make it tolerably clear that mechanisms that modulate postnatal dendritic growth and dendritic branching are not inextricably coupled *in situ*. For example, although the dendritic trees of pyramidal cells in V1 of the macaque were at their largest at 2D, they were most branched at 3 weeks of age. Cells in TEO and posterior TEpd in the macaque attain their greatest branching complexity at 3 weeks and 7 months of age, respectively, yet these cells are characterized by continual dendritic growth from PND2 to adulthood (Elston et al., 2010a). Cells in TEav are most branched in the adult, but attain their greatest size at 7 months of age (Elston et al., 2011b). Moreover, the mechanisms modulating growth and branching may exert their influence for different periods of time in different cortical areas. For example, in V2 the greatest branching complexity was observed in the dendritic trees at 3 weeks of age, whereas in V4 the greatest branching complexity occurred at 3^1^/_2_ months of age (Elston et al., 2010a). In A1 the greatest branching complexity was observed at 7 months of age (Elston et al., 2010b). Growth of the dendritic tree and branching also show a degree of disjoin in the marmoset monkey: pyramidal cells in TE and area 12, in particular, are characterized by a marked increase in branching complexity from 2 months to 4.5 years of age while there is only a modest increase in the size of the trees during this same time (Oga et al., 2013). Recent detailed studies of pyramidal cell development in rodent somatosensory cortex also reveal different temporal profiles for growth, branching and spinogenesis (Romand et al., 2011). The authors found fast growth during the early postnatal period and the slow localized growth at latter ages, which they interpret as supporting functional compartmental development. Indeed, different compartments within the dendritic trees of pyramidal cells were shown to grow independently of each other (Romand et al., 2011).

## Spinogenesis

**Figure 7:**
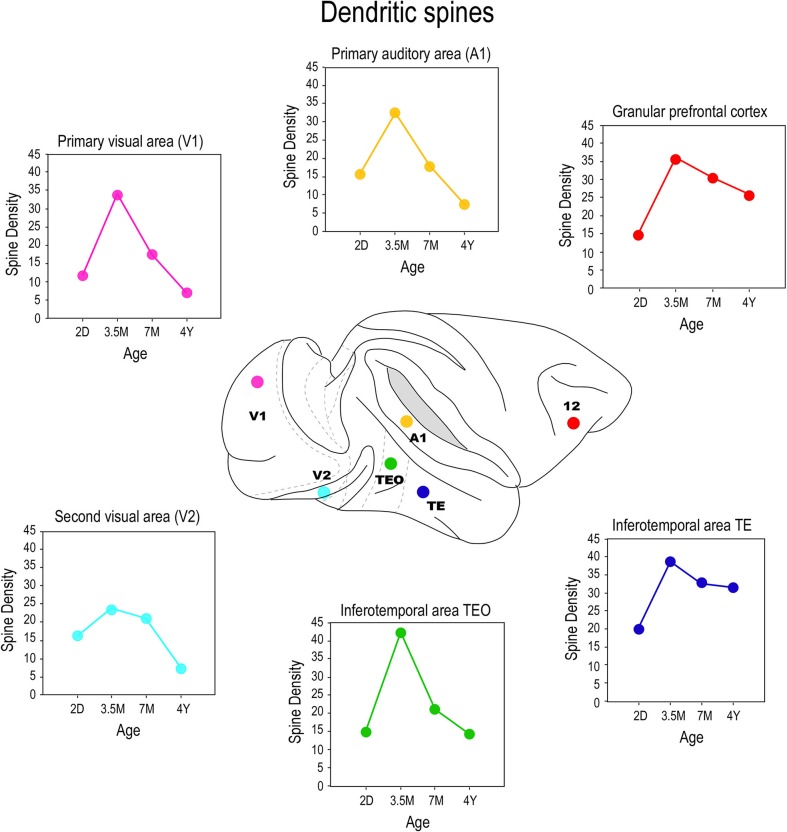
**Spinogenesis**. Plots of the peak spine density (defined as the average of the maximum spine density observed in all 10 μm segments of dendrite from the soma to the distal tips of the dendrites of all cells quantified for any given age) of layer III pyramidal cells sampled in the primary visual area (V1), the second visual area (V2), the primary auditory area (A1), the posterior and anterior portion of the inferior temporal cortex (TEO and TE, respectively) and prefrontal cortex (Brodmann's area 12) at postnatal day two (2D), three and a half months of age (3.5M), seven months of age (7M) and adults (AD). Note that in V1, V2, and A1 there is a net reduction in the number of spines in the dendritic trees of pyramidal cells from birth to adulthood whereas in TEO, TE and area 12 there is a net increase in the number of spines over the same period. That is to say, the total number of spines grown in V1, V2, and A1 following the onset of sensory experience is, on balance, lost with increasing sensory experience, but is stabilized, to varying degrees, with increasing associative and executive function in TEpd, TEav and area 12. Data taken from Elston et al. (2009, 2010a,b, 2011a).

By injecting cells in thick cortical slices, cut in the tangential plane, it is possible to reconstruct the entire basal dendritic trees of individual neurons. Thus, it is possible to draw and tally spines along the entire extent of dendrites, from the cell bodies to the distal tips, and determine the spine density as a function of distance from the soma (Eayrs and Goodhead, 1959; Valverde, 1967; Elston, 2001). Consistent with Pasco Rakic's findings for synapses (1986), we found that maximum peak spine density (defined as the average of the maximum spine density observed in all 10 μm segments of dendrite from the soma to the distal tips of the dendrites of all cells quantified for any given age) was greatest in all cortical areas at approximately 3.5 months (Figure 7). The maximum peak spine density among cells in the different cortical areas were remarkably similar at 3.5 months of age (approximately 30 spines per 10 micrometers). However, the rate of spine loss in the dendritic trees of pyramidal cells in the different cortical areas varies considerably such that those in V1, for example, prune to a maximum peak spine density of approximately 6 spines per 10 micrometers whereas those in the gPFC prune to a peak of approximately 25 spines per 10 micrometers. Likewise, the maximum peak spine density was observed at approximately 3.5 months of age in the marmoset monkey: approximately 30–40 spines per 10 micrometers (Oga et al., 2013; Sasaki et al., 2014).

In previous studies an estimate of the number of synapses per neuron were made at different ages by dividing the density of synapses in the neuropil with the number of neurons (Huttenlocher, 1979, 1990). These calculations made no distinction between asymmetrical and symmetrical synapses, nor between the different neuronal types (pyramidal vs. spiny stellate), nor compartmentalization within the dendritic trees (e.g., apical vs. basal dendrites). By studying the morphology of individual neurons, it is possible to calculate an estimate of the total number of excitatory synapses to that cell by quantifying the number of dendritic spines [i.e., each dendritic spine receives at least one excitatory input (DeFelipe et al., 1988; Petralia et al., 1994a,b,c; Arellano et al., 2007)]. By multiplying the spine density at a given increment along the dendrites with the number of branches at the corresponding distance it is possible to obtain a better estimate of the total number of excitatory synapses received by individual identified neurons.

These calculations revealed that different numbers of spines were grown in the dendritic trees of cells among these cortical areas, and then subsequently pruned. There was a net decrease in the number of spines in the dendritic trees of cells in V1, V2, V4, and A1 from birth to adulthood, whereas there was a net increase in the number of spines in the dendritic trees of cells in TEO, TE and prefrontal cortex area 12 during this same period (Elston et al., 2009, 2010a,b). In other words, pruning exceeds spinogenesis in V1, V2, V4, and A1 (early and middle sensory areas), whereas spinogenesis exceeds pruning in TEO, TE and prefrontal cortex (higher visual cortex and multimodal association cortex), during the normal course of postnatal development. It is worth noting that, on average, layer III pyramidal cells in prefrontal cortex grew 4 times the number of spines in their basal dendritic trees as did those in V1 (15,900 and 3900, respectively). The number of spines then pruned from cells in gPFC into adulthood (7400) equates to >8 times the total number of spines observed in the dendritic trees of pyramidal cells in V1 of the adult (600–900) (Elston et al., 2009). In the marmoset monkey, spinogenesis greatly exceeded pruning in areas TE and gPFC whereas in V1 spinogenesis slightly outweighed pruning in V1 (Oga et al., 2013).

**Figure 8:**
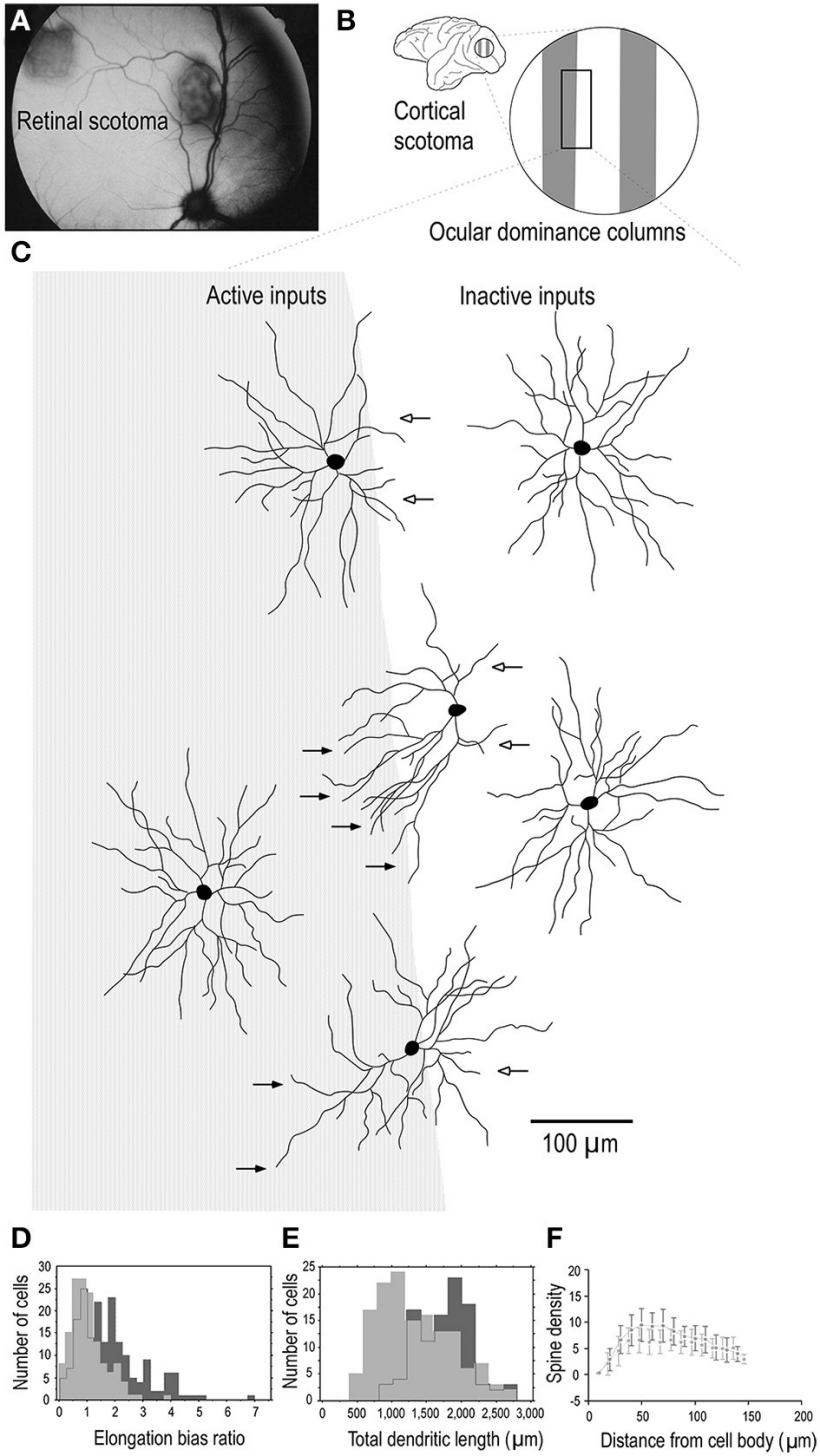
**Adult dendritic growth**. Schematic illustrating placement of a retinal scotoma (laser lesion) **(A)** in the adult macaque monkey, and the corresponding region of the contralateral V1 **(B)**, in which individual neurons were injected across occular dominance columns **(C)**. Cells located near a border of an ocular dominance column appeared to show a regression of dendrites which projected toward the center of the CO-poor column (open arrows) and an elongation of dendrites toward the CO-rich column (closed arrows). Cells located in the centers of the OD columns appeared to be relatively symmetrical. Comparison of over 400 layer III pyramidal cells intracellularly injected in 3 adult monkeys in which we placed monocular retinal lesions, and 5 age/gender/ hemisphere/layer-matched controls, revealed that the basal dendritic trees of layer III pyramidal neurons within the portion of V1 corresponding to the retinal lesion (the lesion projection zone, LPZ) undergo dendritic growth **(D, E)** and spinogenesis **(F)**. Cells in the OD column corresponding to the non-lesioned eye within the LPZ have 65% more spines in their dendritic trees than those in the OD columns corresponding to the lesioned eye. The structural changes are not limited to cells within the LPZ in V1. Neurons in the region of cortex immediately surrounding the LPZ in V1 (the peri-LPZ) are considerably larger and more spinous than those within the LPZ. These cells have double the number of spines in their dendritic trees as compared with those in age/gender/hemisphere/layer-matched controls. Others have also reported perturbations in the peri-LPZ in V1, including marked changes in the expression of receptor subunits (Arckens et al., 2000) and expansion in receptive field sizes (Kaas et al., 1990; Chino et al., 1992; Schmid et al., 1996; Calford et al., 1999). In conjunction, these data suggest that anatomical and functional reorganization in the peri-LPZ differs in magnitude to that observed within the LPZ. Figure modified from Elston and DeFelipe (2002).

One interpretation of these findings may be that all synaptic connections to the dendritic trees of pyramidal cells are formed by 3.5 months of age, some of which are then pruned into adulthood. However, such an interpretation can't account for the presence of dendritic spines throughout the entire dendritic trees of cells that continue to grow in size beyond 3.5 months of age. Nor can it account for the fact that where cells continue to increase in branching complexity beyond the peak in spinogenesis, spines are found studded along all dendrites. A more parsimonious interpretation is that although there may be net reduction in spine density in the dendritic trees of cells from 3.5 months of age into adulthood, new spines are continually grown through this period. What is revealed then in our data is a snapshot of the absolute number of dendritic spines accounting for new spine growth and spine loss. While such spinogenesis, dendrite growth and axogenesis has been demonstrated in the adult cortex following injury (Figure 8; e.g., Jones and Schallert, 1992; Darian-Smith and Gilbert, 1994; Jones et al., 1996; Elston and DeFelipe, 2002; Trachtenberg et al., 2002; Matsuzaki et al., 2004; Knott et al., 2006; Keck et al., 2008; Knott and Holtmaat, 2008; Sala et al., 2008), our data suggest adult spinogenesis is a feature of the normal healthy growing brain, that a percentage of spines are continually turned over, the average number per neuron varying among cortical areas. That is to say, dendrites and spines of pyramidal cells continue to grow throughout the entire life cycle, including infancy, childhood, adolescence, adulthood and senescence, as a normal process without the necessity of occasioning a trigger such as injury to the peripheral or central nervous system. At times there will be a net increase in neurite (dendrite, axon, spine) growth, at times there will be a net reduction in neurite growth.

## Axon projections

**Figure 9:**
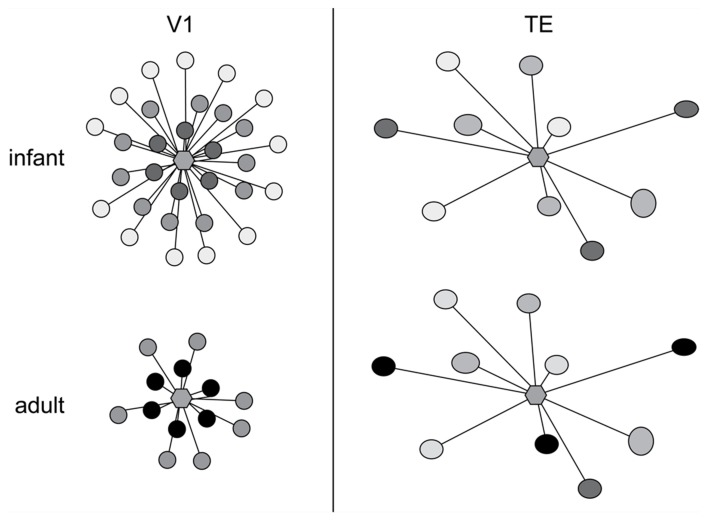
**Axon growth in development**. Schematic illustration of the postnatal development of intrinsic axon patches in the primary visual area (V1) and visual association area TE in the macaque monkey. At center in each representation is a hexagon representing a single injection site. The shaded circles represent the intrinsic patches labeled by the single injection site. The intensity of labeling varied among patches, illustrated as varying shades of gray. Note that labeled patches were observed both in TE and V1 at 1 week of age (infant). Even at this age the size, interpatch distance and distribution pattern differ between the two cortical areas. The patterns of connectivity then diverge, resulting in qualitatively and quantitatively different patterns in the adult. In particular, the number and the maximal lateral extent of patches are reduced during postnatal development in V1, whereas in TE the number of patches remains constant and connectivity among these patches strengthens with aging. Based on data from Wang et al. (1998).

The above data give us some insights into postnatal changes in the postsyanpatic matrix (dendritic spines and dendrites) in cortical circuitry, what then of the development of the presynaptic matrix (axons and axon boutons)? While it has been well documented that presynaptic matrix is characterized by a patchwork or lattice-like structure in the adult cortex, and that the size and pattern of these patches vary among cortical areas (Rockland and Lund, 1982, 1983; Amir et al., 1993; Lund et al., 1993; Fujita and Fujita, 1996; Melchitzky et al., 1998, 2001; Tanigawa et al., 1998, 2005; González-Burgos et al., 2000), little is known of their development. Fujita and colleagues quantified the postnatal development of these axonal patches from birth to adulthood in areas V1 and TE of the macaque monkey (Wang et al., 1998). The axon patches are present at birth (PND7) and follow divergent maturation profiles with the onset of visual experience, with some shared characteristics. Of particular note was the decrease in the number of the patches is observed in V1 from PND7 to adulthood as compared with the trend in area TE where the number of patches remained consistent (Figure 9). There was also a decrease in the horizontal extent of the patches in V1 from PND7 to adulthood as compared with TE where the horizontal extent of axon patch labeling was maintained over the same period.

At the level of individual axons, in both V1 and TE axons within the patches were relatively straight at birth, and developed increasingly more varicosities with increasing postnatal age. In both TE and V1, the density of boutons along axons located within the patches increased with age, at least from infants. These data reveal that axonal branches and boutons within the patches in area TE continue to develop over many months, with connections being strengthened with aging. Previous studies revealed that the horizontal axons form this patchy pattern in V1 of the macaque monkey at least 3 weeks prior to birth (Coogan and Van Essen, 1996). Likewise, ipsilateral (Barone et al., 1996; Coogan and Van Essen, 1996) and callosal (Meissirel et al., 1991; Schwartz and Goldman-Rakic, 1991) corticocortical projections in macaque V1 are initially diffuse and then become patchy before birth. Although little is known of the patches in prenatal TE, and further studies are obviously needed, the maturity of horizontal axons at PND 7 (injection at PND 4) suggests that a patchy projection pattern is also established *in utero* in TE. Thus, patch formation does not likely require visual experience in either the striate cortex or extrastriate cortex, at least, in monkeys (see Schwartz and Goldman-Rakic, 1990, for a review).

## Neuronal physiology: developmental changes and areal specialization

**Figure 10:**
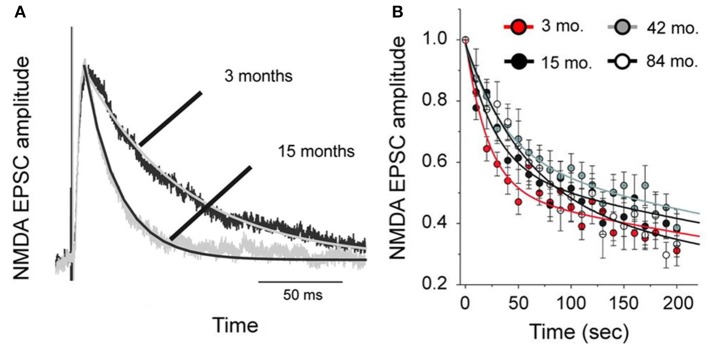
**Age differences in cell physiology**. Plots of the difference in NMDA-mediated excitatory postsynaptic currents (NMDA-EPSCs) in pyramidal cells sampled from the prefrontal cortex of 3 and 15 month old macaque monkeys. **(A)** Examples of average NMDA-EPSCs scaled to the same peak amplitude illustrating the exponential functions fit to the traces. Note the differences in duration. **(B)** The amplitude of NMDA-EPSCs was normalized relative to that of the first response recorded after resuming stimulation in the presence of an NMDA antagonist, MK801. The lines represent double-exponential functions fit to the data from animals of different ages by non-linear regression. Modified from Gonzalez-Burgos et al. (2008).

**Figure 11:**
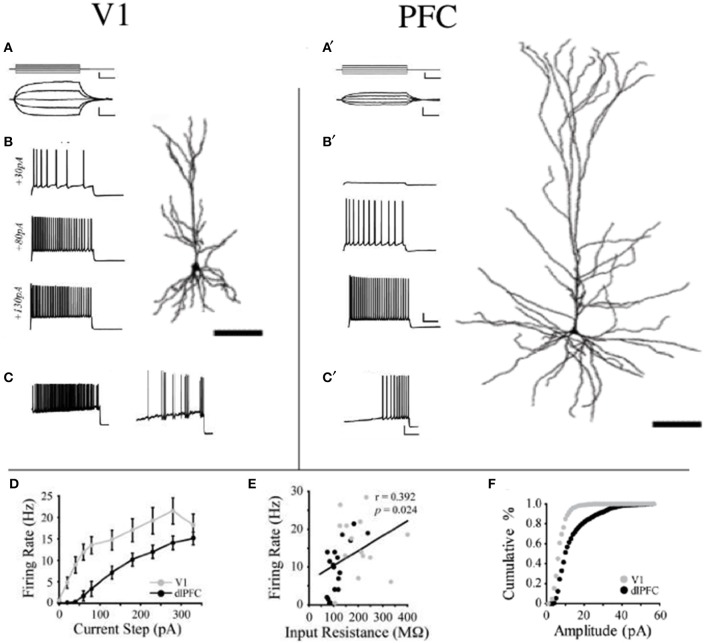
**Area differences in cell physiology**. Reconstructions of layer III pyramidal cells sampled in the adult primary visual area (V1) and granular dorsolateral prefrontal cortex (PFC) and schematics illustrating their voltage responses to **(A,A^1^)** 200-ms current pulses (−40 to +40 pA) and **(B,B^1^)** 2-s current steps of +30, +80, and +130 pA and their firing patterns in response to a current ramp protocol resulting in phasic and tonic discharge **(C,C^1^)**. **(D)** Mean firing rates of V1 vs. PFC neurons are illustrated in response to a series of depolarizing current steps. V1 was significantly different from PFC at steps +20 to +280 pA (*p* < 0.05). **(E)** Relationship between input resistance and firing rate is illustrated in response to a +130 pA current step for all V1 and dlPFC neurons. Linear regression (black line) demonstrates a significant positive correlation. **(F)** Cumulative frequency histograms of sEPSC amplitudes (1 pA bins) in V1 and PFC neurons. Modified from Amatrudo et al. (2012).

To test whether changes in the morphology of pyramidal cells during development outlined above influences cell physiology, Fujita and colleagues made intracellular whole-cell clamp recordings of pyramidal cells in V1 and TE from animals aged 10 days to 6 years of age (Maruyama et al., 2007). A number of physiological properties were quantified, including resting potential, firing threshold, membrane time constant, depolarizing sag, duration of individual action potentials, and spike-frequency adaptation. Most notably, membrane time constant, depolarizing sag, duration of individual action potentials, and spike-frequency adaptation ramped up until 7 months of age in cells in V1, but continued to ramp up over a protracted period of time in cells in TE. By contrast, the depolarizing sag increased through all ages in V1, but did not change until 1 year of age in TE. In addition to these changes in intrinsic membrane properties of individual neurons, synaptic transimission properites also change postnatally. For example, the excitatory postsynaptic currents recorded in layer III pyramidal cells at 15 months of age in the gPFC are considerably different to those recorded at 3 months of age (Gonzalez-Burgos et al., 2008) (Figure 10). These developmental differences in the electrophysiological signatures of pyramidal cells result in different, arguably specialized, functional signatures in the adult cortex. For example, Luebke and colleagues recently demonstrated in an elegant study that pyramidal cells in V1 have a significantly higher input resistance, depolarized resting membrane potential, higher action potential (AP) firing rates, lower spontaneous postsynaptic currents and faster kinetics than those in granular PFC (Amatrudo et al., 2012) (Figure 11). Moreover, pyramidal cells in V1 and TE of the mature brain show a diametrically opposed reaction to extrinsic tetanic stimulation: extracellular stimulation (20–40 pulses at 40–100 Hz, applied every 4 s for 3–5 min) of intrinsic horizontal connections of layers 2 and 3 results in long term depression (LTD) of extracellular potentials in V1 and long term potentiation (LTP) in TE (Murayama et al., 1997). Although Luebke and colleagues were unable to demonstrate such changes in the electrophysiological properties in V1 by *in vitro* recording (Luebke et al., 2013), on balance, we suggest the above data reveal that the anatomical and electrophysiological profiles of pyramidal cells vary among cortical areas at birth, and continue to diverge into adulthood.

Comparison of the results of studies in primates with those in rodents reveals some interesting differences in their electrophysiological properties with aging. For example, whereas resting potential of layer III pyramidal cells remained unchanged in V1 and TE of the macaque monkey, there is a significant change in these same cells in A1 of the mouse (Oswald and Reyes, 2008; Romand et al., 2011). Input resistance of layer III pyramidal cell differed between V1 and TE of the macaque monkey remained unchanged with age. In mouse A1, the input resistance of layer III pyramidal cells decreased with aging (Metherate and Aramakis, 1999; Oswald and Reyes, 2008). Neuronal discharge following an intracellularly applied electrical pulse increased with age in layer III pyramidal cells in mouse A1, as was the case in macaque V1, but decreased in macaque TE. Discharge with increasing age also increased in layer V pyramidal cells in rat prelimbic cortex (Zhang, 2004). Zhang (2004) also reported a decrease in input resistance, resting potential and time constant with aging, as also reported in layer V pyramidal cells in rat visual and somatosensory cortex (McCormick and Prince, 1987; Kasper et al., 1994). Clearly then, the electrophysiological properties of pyramidal cells may vary independently among different cortical layers, different cortical areas and different species.

It is worth remembering that while pyramidal cells vary in size and branching complexity among cortical areas in some rodents (Benavides-Piccione et al., 2002, 2006; Ballesteros-Yáñez et al., 2006; Elston et al., 2006b), the extent of this variation is but a small fraction of that reported in higher primates. For example, in the mouse there is a 2-fold difference in the number of dendritic spines in the basal dendritic trees of layer III pyramidal cells across the adult cortical mantle. In adult primates, there is a 23-fold difference in the number of dendritic spines in the basal dendritic trees of layer III pyramidal cells between V1 and the gPFC (see Elston, 2003a, for a review). Accordingly, differences in the postnatal developmental profiles of pyramidal cell electrophysiology reported in rodents may well to underscore that evident in primates.

## Neuronal receptive field properties in postnatal development

**Figure 12:**
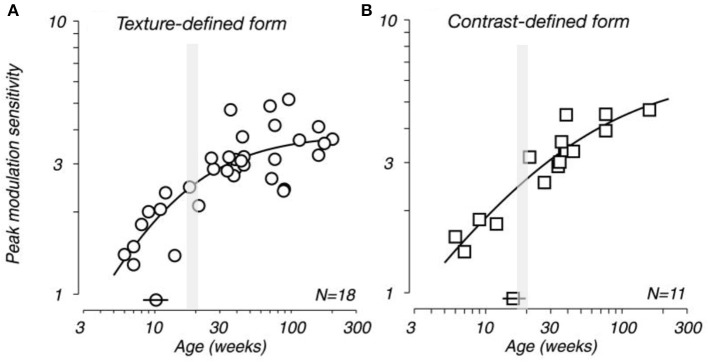
**Developing visual sensitivity**. Plots illustrating the developmental time course of peak behavioral modulation sensitivity for **(A)** texture-defined and **(B)** contrast-defined form as a function of age. Isolated symbols intersected by horizontal bars (above the x-axis) indicate the age at which modulation sensitivity reached half of adult levels (10 and 17 weeks for **A,B**, respectively). *N* indicates the number of monkeys tested. Because many of these subjects were tested longitudinally, each plot contains more data points than the indicated population size. Note that the peak spine and synapse density occurs at approximately 3.5 months of age (gray shading). Modified from El-Shamayleh et al. (2010).

Movshon et al. (1999, 2000) demonstrated that receptive field sizes of neurons located in the central 5 degrees of the visual representation in V1 decrease in size from early postnatal development to adulthood. In addition, they demonstrated that the spatial resolution of cells in V1 (the highest spatial frequency at which cells give a response of at least 10% of its maximum) increases from birth to adulthood. There is also a general trend for increasing surround suppression from birth to adulthood (Kiorpes and Movshon, 2003; Chino et al., 2004) and facilitation develops slowly over the first year after birth (Li et al., 2013). These changes in receptive field size, spatial acuity and surround suppression could plausibly be attributed to changes in the geometrical sampling of neurons as their dendritic trees change shape (Sholl, 1955; Ferster, 1998; Taylor et al., 2000; Taylor and Vaney, 2002). Specifically, as the dendritic trees of neurons decrease in size with maturation they may sample a progressively smaller portion of the visuotopically organized afferent projection and thus have progressively smaller receptive fields. The developmental time course of sensivity to global form and motion and visual texture modulation have been shown to be measured in years, rather than months (Kiorpes and Movshon, 2004; El-Shamayleh et al., 2010; Kiorpes et al., 2012). In conjunction these data suggested a protracted developmental profile for neurons in V1, both morphologically and functionally, that extends well beyond the peak synaptogeneses demonstrated relatively early in development (Figure 12).

Chino and colleagues (Zhang et al., 2008) reported that in V2, receptive fields of neurons mature later than those in V1 neurons, consistent with our finding that the dendritic trees of pyramidal cells in V2 continue to grow from 2D to 3^1^/_2_ months of age while those in V1 become smaller during this time. Although they report that by 4 weeks of age the spatial receptive field structure in V2 is as complex as in adults (Zhang et al., 2013), but their ability to discriminate fine disparity differences was significantly reduced compared with adults (Maruko et al., 2008). V2 neurons of 8-week-old monkeys had significantly lower optimal temporal frequencies and resolutions than those of adults (Zheng et al., 2007). Neurons in area TE do not develop their adult-like response properties until even later in development (Rodman et al., 1993), consistent with our findings of continued growth and branching from birth. Furthermore, anatomical connections of TE undergo a protracted period of refinement from birth to adulthood compared to other visual areas (Webster et al., 1991, 1994, 1995; Rodman and Consuelos, 1994; Barone et al., 1996; Coogan and Van Essen, 1996) and this region becomes myelinated much later in development than V1 (Rodman, 1994). The anatomical data in TEO and TE, which reveal progressively larger dendritic trees from birth to adulthood, parallel the physiological data for increasing receptive field size during postnatal maturation.

In addition to the size of the dendritic trees, their branching structure may influence aspects of cellular function. Considerable work has been done in the retina relating neuronal branching structure to functional qualities such as direction selectivity (see Vaney et al., 2012, for a review). Although less well studied, it has been proposed that dendrtic branching structure may be important in V1, for example, in determining orientation and direction selectivity (Pettigrew, 1974; Tieman and Hirsch, 1982; Elston and Rosa, 1997; Ferster, 1998; Livingstone, 1998). Moreover, the onset of form discrimination at 3 weeks of age (Zimmermann, 1961) occurs at the time when cells are most branched. Could the increase in the number of branches facilitate compartmentalization of processing within the dendritic tree (Poirazi and Mel, 2001; Chklovskii et al., 2004) and allow detection of inputs associated with asymmetric features throughout the dendritic tree?

## Specialized pyramidal cell growth profiles result in specialized cortical circuits in the mature brain

**Figure 13:**
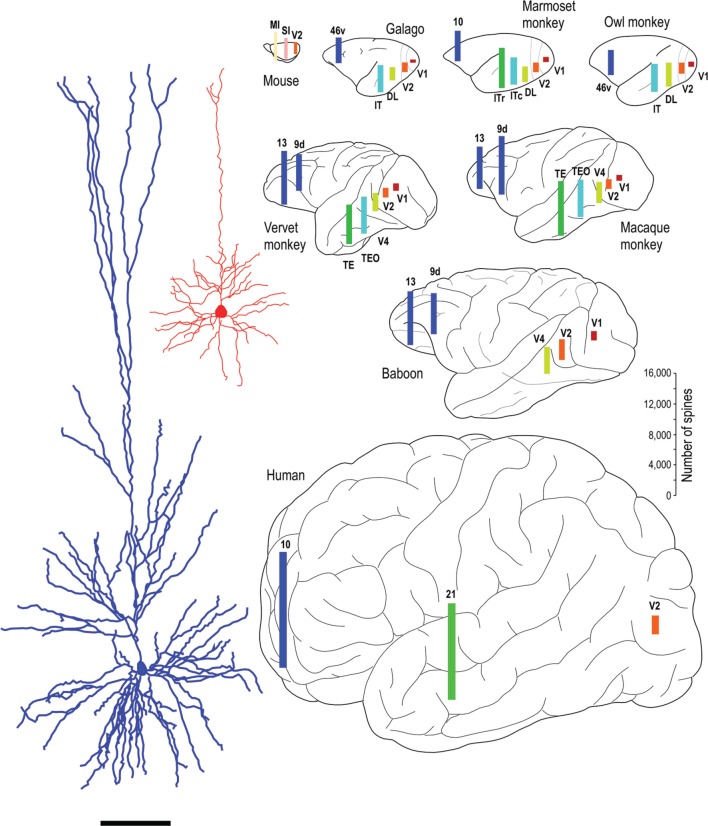
**Specialization in pyramidal cell structure in the adult brain**. Schematic illustrating regional and species differences in pyramidal cell structure. At left are illustrated two individual layer III cells sampled from the primary visual area (V1) and the inferotemporal cortical area TEpd of the adult macaque monkey. Note the difference in thickness of layer III between the two cortical areas, the tangential extent of both the basal and apical dendritic trees. Illustrated at right are the estimates of the total number of spines in the basal dendritic trees of layer III pyramidal cells in visual areas V1, V2, V4 (DL), TEO (ITc), TE (ITr), and prefrontal cortex (areas 9d, 10, 13, and 46v of Preuss and Goldman-Rakic, 1991). Prefrontal cortex refers as originally defined by Brodmann (1913) as granular cortex anterior to the central sulcus (see Elston and Garey, 2004, for a translation). Bars are color coded for homology, that is, homologous/analagous cortical areas are illustrated by a single color. The bars representing the number of spines are not necessarily located in the exact position from which the cells were sampled, but have been positioned for aesthetic balance. Schematics of the different brains are not drawn to scale. Data taken from the mouse (Ballesteros-Yáñez et al., 2006), galago (Elston et al., 2005b,c), owl monkey (Elston, 2003b,c), marmoset monkey (Elston et al., 1999b, 2001), vervet monkey (Elston et al., 2005d, 2011b), macaque monkey (Elston and Rosa, 1997, 1998; Elston et al., 1999a, 2001, 2011b; Elston, 2000), baboon (Elston et al., 2005e, 2011b) and human (Elston et al., 2001). Systematic differences have also been demonstrated in the total number of spines in the basal dendritic trees of layer III pyramidal cells in sensorimotor and cingunalte cortex in all species studied to date, including the adult vervet monkey, macaque monkey and baboon (Elston and Rockland, 2002; Elston et al., 2005a,f,g,h,i) (not illustrated).

**Figure 14:**
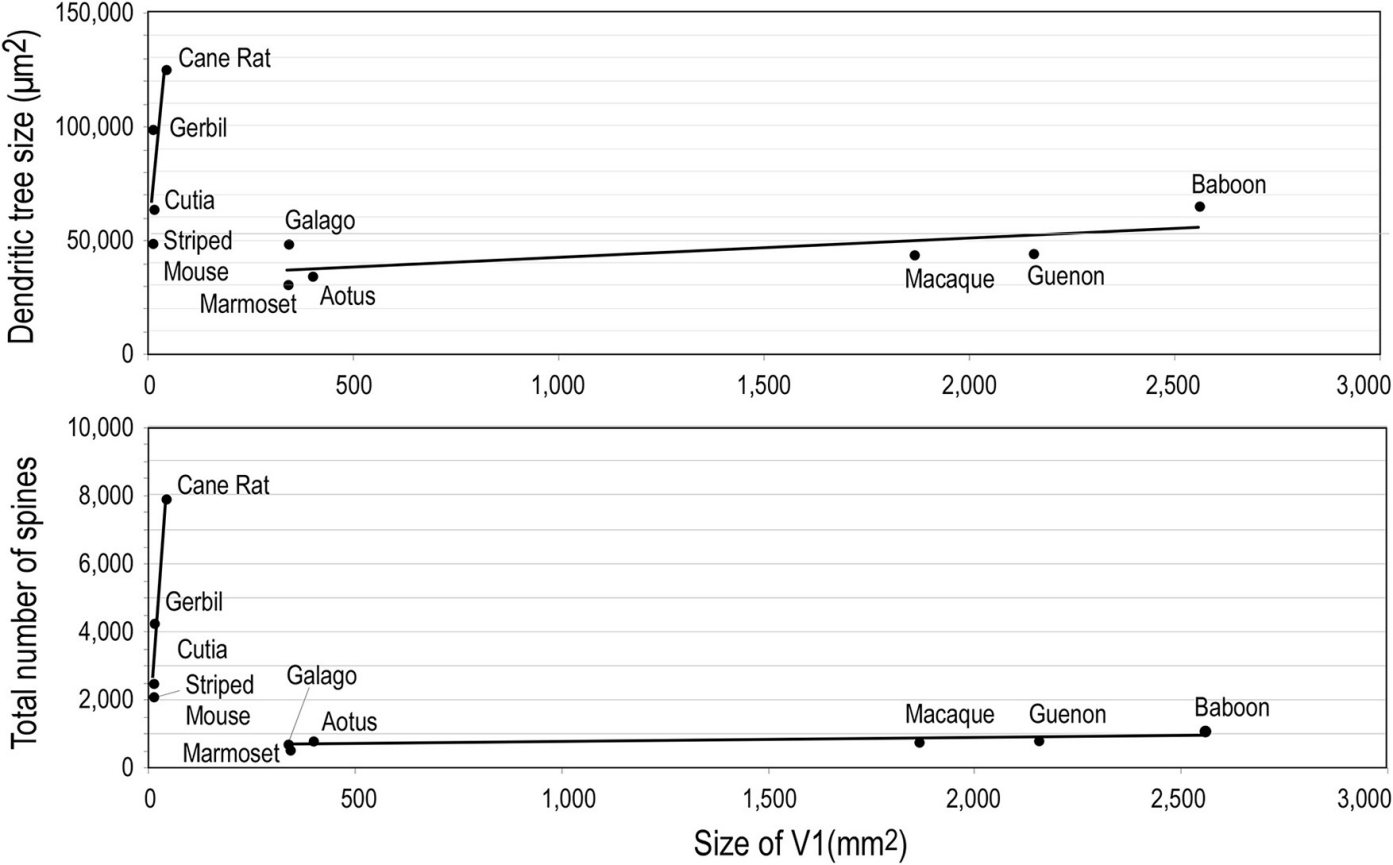
**Neuron growth and cortical size**. Graphs illustrating phylogentic differences in the trends between pyramidal cell structure and size of the of the primary visual area (V1) in rodents and primates. Illustrated are the size of, and number of dendritic spines in, the “average” pyramidal cell vs. the size V1 in the cane rat, bushvelt gerbil, striped mouse, agouti (cutia), tree shrew, galago, marmoset monkey, owl monkey, guenon (vervet monkey), macaque monkey, and baboon. Separate trend lines (linear regressions) are illustrated for the rodent and primate data (reproduced from Elston and Manger, 2014).

Whatever the explanation, the different developmental profiles result in marked differences in pyramidal cell structure among cortical areas in the mature brain (Figure 13). Cells become increasingly larger and more spinous with anterior progression through visual areas V1, V2, V4, TEO, and TE (Elston and Rosa, 1997, 1998; Elston et al., 1999b, 2005b,d,e; Elston, 2003b). Likewise, cells become increasingly larger and more spinous with progression through somatosensory areas 3b, 5, and 7 (Elston and Rockland, 2002; Elston et al., 2005h,i). Those in polysensory cortex, such as the superior temporal polysensory area (STP) are larger and more spinous than those in TE (Elston et al., 1999a). Those in cingulate cortex are larger and more spinous than those in sensory and sensory association cortex (Elston et al., 2005a,f,g), and those in prefrontal cortex are among the most spinous of all pyramidal cells in the cerebral cortex (Elston et al., 2001, 2011b; Jacobs et al., 2001; Bianchi et al., 2012). It has also been demonstrated that pyramidal cells are increasingly more complex in structure and more spinous in primate species with increasingly larger granular prefrontal cortex (Elston et al., 2005c, 2006a, 2011b). Pyramidal cells in the adult brain of rodents and primates appear also to be influenced by different developmental and phylogenetic controls (Elston and Manger, 2014). Pyramidal cells in V1 of adult rodents vary considerably among species, being larger, more branched and more spinous with increasing size of V1, whereas that in adult primate V1 is relatively constant, irrespective of the size of V1 (Figure 14).

**Figure 15:**
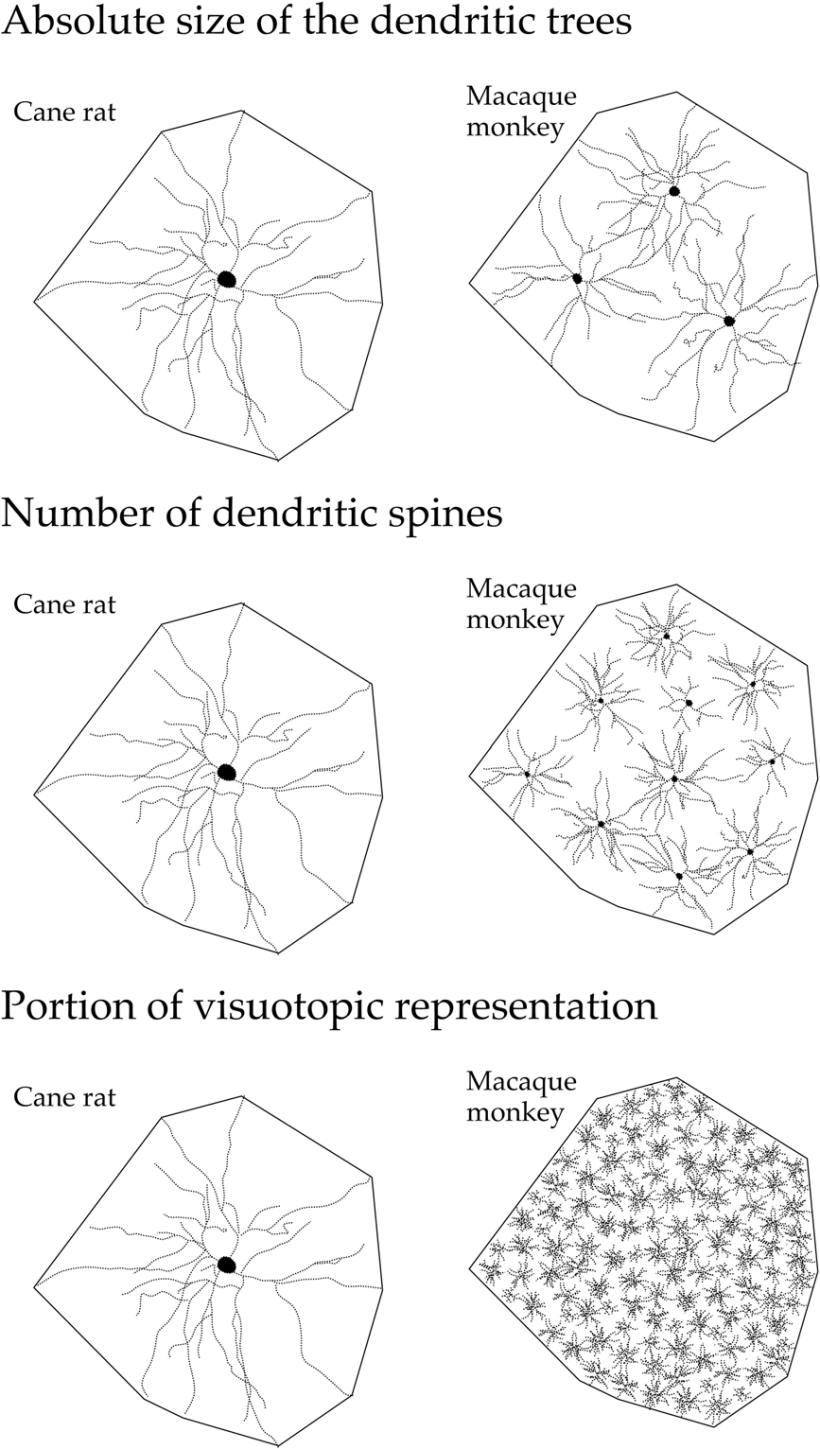
**Neuron structure and cortical connectivity**. Schematic, not drawn to scale, illustrating the dendritic tree of the “average” layer III pyramidal cell in V1 of the adult cane rat and macaque monkey: that in the cane rat is, on average, three times the size of that in the macaque monkey. Differences in the size, branching structure and spine density result in a more than 10-fold difference in the estimate of the total number of spines, putative excitatory inputs, in the basal dendritic tree of the “average” layer III pyramidal cell between the two species. As V1 in the adult macaque is, on average, more than 40 times the size of V1 in the adult cane rat, the dendritic trees of cells in the adult cane rat sample a portion of the visuotopic field approximately 120 times larger than do those in the adult monkey. Thus, 120 cells are required in the macaque monkey V1 to achieve the spatial coverage of a single cell in the cane rat. One hundred and twenty cells in the adult macaque V1 have, on average, a sum total of more than 88,000 spines (120 × 743), being more than 110 times the number of spines in an individual cell in the adult cane rat. (reproduced from Elston and Manger, 2014).

These structural differences potentially influence the number of inputs received by pyramidal cells in any given cortical area, the manner in which these inputs are integrated within the dendritic trees, and the degree of interconnectivity between neurons within cortical circuits (Jacobs and Scheibel, 2002; Elston, 2003a, 2007; London and Häusser, 2005; Spruston, 2008). In V1, the differences in the size of the dendritic trees, in the tangential plane, influence the portion of the visutopic representation sampled by individual cells (Figure 15). Both the functional capacity of neurons and their potential for plastic change have been correlated with their morphological complexity (Poirazi and Mel, 2001; Chklovskii et al., 2004). Moreover, the different structural complexity of pyramidal cells in sensory, association and executive cortex may provide an anatomical platform for tonic vs. phasic sampling, believed to be important for memory (Miller and Cohen, 2001; Funahashi and Takeda, 2002; Elston, 2007).

In addition, differences have also been reported in inhibitory circuitry among cortical areas in the adult brain. Specifically, the density and connectivity of interneurons may differ among cortical areas (Hof and Nimchinsky, 1992; Kritzer et al., 1992; Kondo et al., 1994; Hof and Morrison, 1995; DeFelipe et al., 1999; Elston and González-Albo, 2003; Yáñez et al., 2005; Benavides-Piccione and DeFelipe, 2007; Freire et al., 2010, 2012). The different types of interneurons, such as Chandelier cells, Martinotti cells and basket cells, project to different compartments of pyramidal cells, including the soma, axon initial segment and dendrites (see DeFelipe, 1993; Somogyi et al., 1998; DeFelipe et al., 2002 for reviews). The differences in both the density and distribution of interneurons, and pyramidal cell structure, among cortical areas result in quantifiably different patterns of connectivity (DeFelipe et al., 1999; Elston et al., 1999d; Blazquez-Llorca et al., 2010; da Rocha et al., 2012). In future studies it will be of particular interest to determine the developmental profiles of inhibitory neurons (e.g., Anderson et al., 1995), especially in view of the different types of neuronal migration during development: radial and tangential (see Rakic, 1990; Nakajima, 2007; Jones, 2009; Chédotal, 2010; Govek et al., 2011 for reviews). It will also be of great importance to expand the scope of research to identify possible species differences in postnatal development of cortical circuitry, particularly in human prefrontal cortex which is disproportionately large compared with that in other primates (Brodmann, 1913) translated by Elston and Garey (2004), to better comprehend the development of the neural substrate for cognitive functions (see Fuster, 1997; Goldman-Rakic, 1999; Preuss, 2000; Elston, 2007; Rakic, 2009 for reviews).

## Conclusions

Recent cell injection studies reveal variation in the developmental profiles of pyramidal cells among different cortical areas in the primate brain. The different growth profiles of pyramidal cells among cortical areas potentially influence the number of inputs received by neurons in any given cortical area, the manner in which these inputs are integrated within the dendritic trees, and the degree of connectivity between neurons. Together, these features may influence the physiological properties of the cells, and the circuitry they compose. Moreover, the data reveal that spine loss in the developing cortex does not result solely from the pruning of existing spines observed at the peak (approximately 3 months of age) but, rather, by the net reduction of existing and newly acquired spines (both those formed by 3 months of age and in the more mature cortex). Furthermore, the rate of spine loss in the dendritic trees of pyramidal cells varies among cortical areas in both absolute and relative terms. The “use it or lose it” principle may belie the diversity of mechanisms that drive synapse stabilization or elimination in the developing and mature cerebral cortex. Use it but you may still lose it may prove to be just as, if not more, prevalent in the developing brain.

## Funding

This work was supported by an RD Wright Fellowship from the National Health and Medical Research Council of Australia (210341: GNE) and grants from the Japan Science and Technology Agency (Core Research for Evolutional Science and Technology), the Ministry of Education, Science, Sports, and Culture (Japan; grant number 17022025), Osaka University, and the I Hear Innovation Foundation (Australia) and the Sunshine Coast Regional Council (Australia).

### Conflict of interest statement

The authors declare that the research was conducted in the absence of any commercial or financial relationships that could be construed as a potential conflict of interest.
